# Relapse and post-discharge body composition of children treated for acute malnutrition using a simplified, combined protocol: A nested cohort from the ComPAS RCT

**DOI:** 10.1371/journal.pone.0245477

**Published:** 2021-02-03

**Authors:** Natasha Lelijveld, Eunice Musyoki, Susan Were Adongo, Amy Mayberry, Jonathan C. Wells, Charles Opondo, Marko Kerac, Jeanette Bailey

**Affiliations:** 1 No Wasted Lives, Action Against Hunger, London, United Kingdom; 2 Emergency Nutrition Network, Oxford, United Kingdom; 3 International Rescue Committee, Nairobi, Kenya; 4 Childhood Nutrition Research Centre, UCL Great Ormond Street, Institute of Child Health, University College London, London, United Kingdom; 5 Department of Medical Statistics, London School of Hygiene and Tropical Medicine, London, United Kingdom; 6 Department of Population Health, Centre for Maternal, Adolescent and Child Health (MARCH), London School of Hygiene and Tropical Medicine, London, United Kingdom; 7 International Rescue Committee, New York City, New York, United States of America; Addis Ababa University, ETHIOPIA

## Abstract

**Introduction:**

Severe and moderate acute malnutrition (SAM and MAM) affect more than 50 million children worldwide yet 80% of these children do not access care. The Combined Protocol for Acute Malnutrition Study (ComPAS) trial assessed the effectiveness of a simplified, combined SAM/MAM protocol for children aged 6–59 months and found non-inferior recovery compared to standard care. To further inform policy, this study assessed post-discharge outcomes of children treated with this novel protocol in Kenya.

**Methods:**

Six ‘combined’ protocol clinics treated SAM and MAM children using an optimised mid-upper arm circumference (MUAC)-based dose of ready-to-use therapeutic food (RUTF). Six ‘standard care’ clinics treated SAM with weight-based RUTF rations; MAM with ready-to-use supplementary food (RUSF). Four months post-discharge, we assessed anthropometry, recent history of illness, and body composition by bioelectrical impedance analysis. Data was analysed using multivariable linear regression, adjusted for age, sex and allowing for clustering by clinic.

**Results:**

We sampled 850 children (median age 18 months, IQR 15–23); 44% of the original trial sample in Kenya. Children treated with the combined protocol had similar anthropometry, fat-free mass, fat mass, skinfold thickness z-scores, and frequency of common illnesses 4 months post-discharge compared the standard protocol. Mean subscapular skinfold z-scores were close to the global norm (standard care: 0.24; combined 0.27). There was no significant difference in odds of relapse between protocols (SAM, 3% vs 3%, OR = 1.0 p = 0.75; MAM, 10% vs 12%, OR = 0.90 p = 0.34).

**Conclusions:**

Despite the lower dosage of RUTF for most SAM children in the combined protocol, their anthropometry and relapse rates at 4 months post-discharge were similar to standard care. MAM children treated with RUTF had similar body composition to those treated with RUSF and neither group exhibited excess adiposity. These results add further evidence that a combined protocol is as effective as standard care with no evidence of adverse effects post-discharge. A simplified, combined approach could treat more children, stretch existing resources further, and contribute to achieving Sustainable Development Goal Two.

## Introduction

Severe and moderate acute malnutrition (SAM and MAM) affect more than 50 million children worldwide and result in increased risk of illness, reduced physical and mental development, and death [1, 2]. While treatment for SAM has made huge strides in the past decade, with the introduction of ready-to-use therapeutic food (RUTF) and community management (CMAM), there is currently no consensus on how best to manage children with MAM. The lack of evidence in this area has prevented development of international guidelines, hence urgent research is needed. In addition, treatment coverage for both SAM and MAM remains low, with at least 80% of children 6–59 months old with acute malnutrition not accessing care [3]. In many settings, children with MAM receive no support, or they receive ready-to-use supplementary food (RUSF), which is dependent on a dedicated team to deliver this service and an independent supply chain [4, 5]. One option to improve the reach of treatment services, as well as potentially improve outcomes for MAM children, is combining the treatment of SAM and MAM into one protocol, using one ready-to-use food product.

The ComPAS trial (Combined Protocol for Acute Malnutrition Study) was a single-blinded, cluster randomised, controlled, non-inferiority trial to compare recovery rates of a combined protocol for uncomplicated SAM and MAM in children 6–59 months in South Sudan and Kenya against the standard treatment protocols in each country [6, 7]. It used a simplified dosage scheme based on mid-upper arm circumference (MUAC) and provided ready-to-use food (RUTF) for both SAM and MAM patients. The simplified dosage provided 2 sachets of RUTF per day for SAM children and 1 sachet per day for MAM children; this was often a lower dosage than what was provided by the standard weight-based dosage calculations for SAM. The combined protocol had non-inferior recovery rates compared to standard treatment (76.3% vs 73.5%), as well as a reduced overall economic cost [8].

To better inform future policy, there is also a need to understand post-discharge outcomes for children treated with a simplified, combined protocol. Outstanding questions include: (1) Do children treated with the combined protocol have comparable sustained recovery and risk of post-discharge morbidity? (2) Are MAM children treated with RUTF rather than RUSF at increased risk of excess adiposity? It is possible that the provision of RUTF could promote the accretion of fat mass rather than lean mass, potentially leading to excess adiposity, which might track into adulthood. This is particularly important to rule out given the rise of non-communicable disease in low-income countries and concerns over the “double burden” of malnutrition [9, 10]. (3) Does the reduced dosage of RUTF for SAM patients in the combined protocol affect the relapse rate after treatment? There have been few studies conducting post-discharge follow-up of acute malnutrition survivors [11], especially for MAM treatment. Hence our current understanding of predictors of post-discharge relapse, in general, is also limited [12].

This study aimed to answer the above questions by following-up children treated for SAM and MAM with either the standard protocol or a novel combined protocol, four months post-discharge in Nairobi, Kenya.

## Methods

### Trial design

This was a follow-up study for a subset of participants in the ComPAS trial; the methods and results of which have been described elsewhere [6, 8] (registered protocol at ISRCTN (ISRCTN30393230)). Briefly, the intervention arm of the trial treated all uncomplicated children whose MUAC was <12.5cm with RUTF: those with MUAC < 11.5cm and/or mild or moderate oedema (+/++) received 2 sachets of RUTF per day and those with MUAC between 11.5 and <12.5cm (and no oedema) received 1 sachet of RUTF per day. The control arm received the standard weight-based dose of RUTF for SAM and RUSF for MAM. Ethical approval for this follow-up study was granted as an amendment to the original study approval by the London School of Hygiene and Tropical Medicine (reference 11826) and the Kenya Medical Research Institute (reference non-KEMRI 551). Informed, written consent from a parent or guardian was required prior to participation in the study.

### Setting

The original trial took place in 12 clinics in Kenya and 12 in South Sudan. This follow-up was only conducted for participants in the Kenya clusters. This was due to logistical constraints in South Sudan of following-up patients in a highly mobile population, as well as vast distances between communities and lack of mobile phone infrastructure. The Kenyan setting was urban health clinics in three sub-counties of Nairobi: Embakasi North, Embakasi East and Embakasi West.

### Participants

Participants comprised surviving children who had been treated for uncomplicated SAM or MAM, defined as mid-upper arm circumference <12.5cm and/or presence of nutritional oedema (+/++), at any of the 12 participating health clinics, and who attended a follow-up appointment four months post-discharge. Children who presented after 6 months post-discharge were not included. To be eligible for treatment, children had to be between the ages of 6–59 months at admission, hence they could be between 10–63 months of age at the time of follow-up.

### Study procedures

At discharge from treatment, caregivers were given an appointment date for their four month follow-up. If participants failed to attend this appointment, a community health worker telephoned the participant, if possible, or attempted to find the participant at home, and encouraged them to attend. Once at their appointment, anthropometry and body composition were measured and a questionnaire completed (see questionnaire in S1 File). Any children who were found to have relapsed into SAM or MAM were readmitted for standard treatment. Any children who relapsed prior to their four-month appointment and sought care were recorded as a relapse case and still measured at four-months post the original discharge date.

### Outcomes

Anthropometric assessments (weight, height, MUAC, oedema) were done following standard WHO procedures and were subject to quality control, which involved a trained study team member taking two readings within an allowable difference [13]. Weight for height (WHZ), weight for age (WAZ), and length for age (LAZ) z-scores were computed using the WHO 2006 growth standards [14]. Relapse was defined as SAM cases that relapsed back to SAM, or MAM cases that relapsed back to GAM (“global acute malnutrition”, i.e. either MAM or SAM).

Bioelectrical Impedance Analysis (BIA) was used to estimate fat and fat-free mass using a BodyStat^™^ 1500 measuring at 50khz (Bodystat, Douglas, Isle of Man) [15]. Two consecutive readings were taken for each child; readings that were not within 10 ohms of each other were repeated after checking the child’s position. Only repeatable BIA measures (<10 ohms difference in impedance) were included in analysis. Height-adjusted vectors (resistance index (R/H) and reactance index (Xc/H)) were computed using the approach of Piccoli et al., [16], while raw impedance (Z) was divided by the square of height to give the impedance index, from which fat-free mass (FFM) values (kg) were predicted using a calibration equation derived from healthy children aged 3 to 18 months in The Gambia [17]. A second equation, from malnourished children in Ethiopia aged 6 months to 14 years, was also used as a check, and yielded similar values [18]. Fat mass (FM) was calculated as the difference of FFM and weight. Phase angle was measured as a composite marker of cell mass and cellular health [19].

Subcutaneous fat levels and fat distribution were measured using skinfold thickness at the tricep and subscapular sites (Holtain Tanner/Whitehouse callipers, Holtain Ltd, Pembrokeshire, UK), representing peripheral and core body fat stores. Measurements were converted to z-scores for age and sex using the WHO 2006 growth standards [14]. Skinfold thickness ratios (subscapular/tricep) were calculated to represent peripheral vs central adiposity.

Food insecurity was assessed by the FAO Food Insecurity Experience Scale (FIES), an 8-question questionnaire which has been widely validated [20]. Other questions included those about any common morbidities suffered by the child in the past week and past 4 months, as reported by the caregiver (see questionnaire in S1 File).

### Sample size

The ComPAS trial in Kenya enrolled 1,973 eligible children. Allowing for 30% loss to follow-up and excluding children discharged after February 2018 (due to logistical constraints), our potential sample size was approximately 840 children for this study. A previous study using BIA [21], reported mean resistance indices of 567 ohm/m (SD 130) and 603 ohm/m (SD 105) in SAM survivors and control children. We estimated that a sample of 840 children across 12 clusters would be sufficient to detect 36 ohm/m difference in Resistance index (R/H) based on mean values in that previous study, with 90% power at the 5% level of significance, and assumed intra-cluster correlation coefficient of 0.05, based on a previous trial [22].

### Statistical analysis

We used hierarchical, multivariable regression analysis in Stata v14 software (StataCorp LP, College Station, Texas USA) to assess differences in continuous outcome data between combined protocol and control arms (anthropometry and body composition). Age and sex were adjusted for in the regression model, as well as accounting for clustering at clinic level. Differences in proportion of children with morbidities post-discharge were compared using logistic regression, accounting for clustering. The odds of relapsing for the combined protocol children vs standard protocol children, and for children with various potential risk factors (including food security status, age, sex, MUAC at admission, MUAC at discharge, weight gain during treatment) was assessed using univariate and multivariable logistic regression. As well as comparing combined protocol to standard protocol, results were disaggregated by SAM and MAM status at treatment enrolment. This was due to differences in treatment protocol for SAM and MAM children; namely, MAM combined-protocol children were treated with RUTF, whereas MAM standard-protocol children received RUSF. Both arms treated SAM children with RUTF, but the dosage was lower on average for the combined protocol vs the standard protocol (105 vs 148 sachets to recovery (MUAC>12.5cm)); 81% in SAM cases received a reduced dosage compared to standard care [8].

## Results

**Fig 1:**
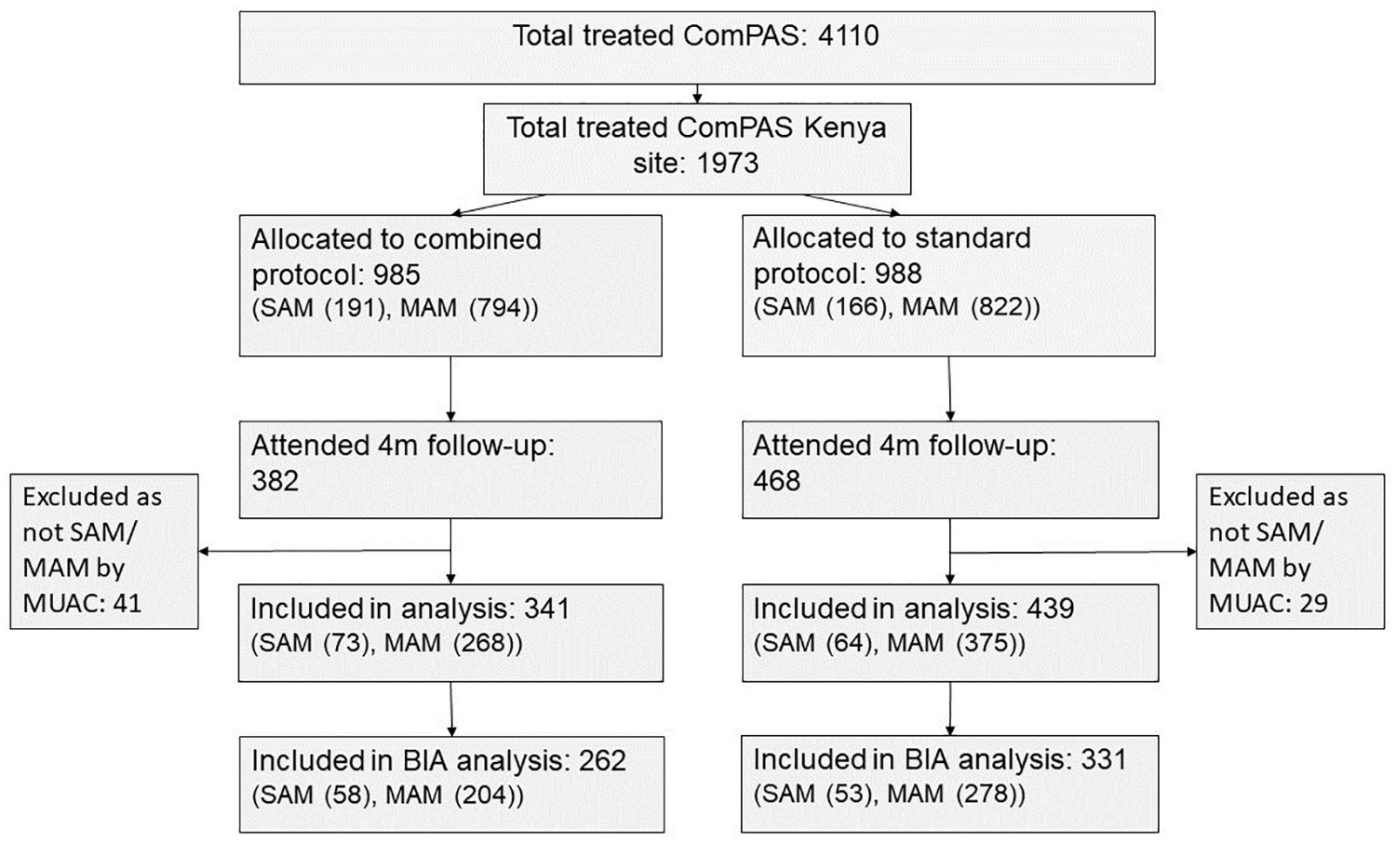
Recruitment flow diagram.

We recruited 850 children into the four-month follow-up study, which met our sample size requirements and was 43% of the original trial sample for ComPAS in Kenya (Fig 1). Children lost to follow-up were similar at baseline to those in our sample, except that this sample had fewer children with oedema and does not represent the small (2%) who died during treatment (S1 Table in S1 File). Children who were SAM based on weight-for-height z-score but not MUAC were excluded from the analysis. Due to several elections and political unrest during the implementation period of the ComPAS trial, many participants moved back to rural areas and were unable to attend their follow-up appointment [8]. Table 1 presents the demographic data for the follow-up sample disaggregated by study arm and SAM/MAM admission status.

**Table 1:** Demographic information for the follow-up sample.

	Combined protocol (N = 6, n = 382)			Standard protocol (N = 6, n = 468)		
	Total	SAM	MAM	Total	SAM	MAM
		n = 73	n = 268		n = 64	n = 375
Median age at follow up (months)	18 (IQR 15–23)	17.5	18	18 (IQR 15–23)	18	18
Males	38%	37%	37%	42%	36%	43%
HIV positive	1%	1%	1%	0%	0%	0%
Weight at admission (kg)	6.9 (1.4)	5.9 (0.9)	6.9 (1.2)	7.1 (1.2)	6.0 (0.7)	7.2 (1.3)
Height at admission (cm)	70.4 (7.6)	66.8 (4.7)	70.0 (6.7)	70.7 (6.7)	66.2 (4.6)	70.9 (6.1)
MUAC at admission (cm)	12.0 (0.6)	11.1 (0.5)	12.1 (0.2)	12.0 (0.5)	11.2 (0.4)	12.1 (0.2)
Oedema at admission	0.8%	-	-	0.2%	-	-
WHZ at admission	-2.32 (0.8)	-3.01 (0.8)	-2.12 (0.7)	-1.91 (0.9)	-2.42 (0.9)	-1.77 (0.8)
WAZ at admission	-2.69 (0.9)	-3.39 (0.9)	-2.53 (0.8)	-2.29 (0.9)	-3.11 (0.9)	-2.14 (0.8)
LAZ / HAZ at admission	-1.85 (1.2)	-2.16 (1.2)	-1.79 (1.3)	-1.65 (1.4)	-2.31 (1.3)	-1.53 (1.5)
MUAC at discharge (cm)	13.0 (0.5)	12.8 (0.7)	12.9 (0.4)	13.0 (0.5)	12.6 (0.7)	13.0 (0.5)
Weight gain (g/kg/day)	2.17 (1.3)	2.80 (1.2)	2.00 (1.2)	1.79 (1.3)	2.06 (1.4)	1.73 (1.3)

*N = number of clusters; n = number of children. Data is presented as mean (SD) unless % is stated or age, which is median (IQR). WHZ = weight for height z-score; WAZ = weight-for-age z-score; LAZ = length for age z-score. MUAC = mid-upper arm circumference. LAZ used for <24 months, HAZ used for ≥24 months.

At admission, in this sub-sample, children in the combined protocol clinics in Kenya were significantly more wasted (WHZ *p*<0.0001), underweight (*p*<0.0001) and stunted (*p* = 0.042) than children in the standard protocol clinics (Table 1). Mean MUAC at admission was similar between the two groups (p = 0.053). The cohort of children in the combined protocol had a greater proportion of females than the standard protocol, and had slightly greater proportion of oedematous SAM cases.

**Fig 2:**
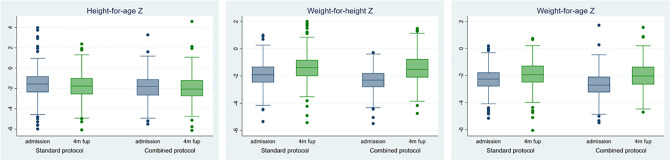
Anthropometry at admission and four-month follow for those in the standard and those in the combined treatment protocol.

Comparing anthropometry between the two protocols at four-month follow-up (Table 2), weight, height, MUAC, WHZ and WAZ were similar between the two groups at this point. This was true for the groups as a whole, and when disaggregated by SAM and MAM children at admission. There was also no statistical difference in LAZ at follow-up between the two protocols when comparing both groups as a whole, nor when comparing SAM admissions. However, there was a difference in LAZ when comparing the MAM children in the standard protocol to the MAM children in the combined protocol (Table 2). When comparing the mean change in LAZ for children between admission and 4-month follow-up, there was no significant difference between the protocols (change in LAZ since admission, MAM standard vs MAM combined, coefficient = -0.02, 95% CI -0.20 to 0.16, *p* = 0.83) (Fig 2).

**Table 2:** Anthropometry at four-month follow-up for combined vs standard protocol, disaggregated by SAM and MAM status.

	SAM cases				MAM cases			
	Standard protocol	Combined protocol	Adjusted Difference	P value	Standard protocol	Combined protocol	Adjusted Difference	P value
	mean (SD)	mean (SD)	(95% CI)*		mean (SD)	mean (SD)	(95% CI)*	
	n = 64	n = 73			n = 375	n = 268		
Weight (kg)	8.1 (1.2)	8.5 (1.1)	0.24 (-0.1, 0.6)	0.18	8.7 (1.1)	8.6 (1.3)	-0.15 (-0.4, 0.1)	0.29
Weight change since admission (kg)	2.1 (1.0)	2.6 (0.9)	0.44 (0.0, 0.9)	0.04	1.5 (0.7)	1.7 (0.8)	0.2 (0.0, 0.4)	0.02
Weight change since discharge (kg)	0.9 (0.6)	0.9 (0.7)	0.01 (-0.2, 0.3)	0.94	0.8 (0.6)	0.9 (0.6)	0.07 (-0.0, 0.2)	0.22
Height (cm)	74.4 (4.4)	75.4 (3.8)	0.49 (-0.7, 1.7)	0.43	77.2 (5.5)	76.6 (6.3)	-0.85 (-2.1, 0.4)	0.20
MUAC	13.0 (1.1)	13.2 (0.9)	0.14 (-0.3, 0.6)	0.53	13.4 (0.7)	13.3 (0.7)	-0.10 (-0.3, 0.1)	0.41
WHZ	-1.55 (1.3)	-1.25 (1.3)	0.18 (-0.3, 0.6)	0.44	-1.37 (0.9)	-1.40 (0.94)	-0.01 (-0.2, 0.2)	0.90
WAZ	-2.30 (1.2)	-2.00 (0.9)	0.13 (-0.2, 0.5)	0.50	-1.84 (0.9)	-2.01 (0.9)	-0.20 (-0.4, 0.03)	0.09
LAZ	-2.36 (1.2)	-2.17 (1.2)	0.04 (-0.4, 0.5)	0.84	-1.71 (1.2)	-1.99 (1.3)	-0.35 (-0.7, -0.02)	0.04

n = number of children.

*adjusted for age and sex, allowing for clustering. Unadjusted weight is significantly smaller in standard arm due to younger age, difference is not present after adjusting. WHZ = weight for height z-score; WAZ = weight-for-age z-score; LAZ = length for age z-score. MUAC = mid-upper arm circumference.

When comparing morbidity outcomes at the four-month follow-up for the standard vs combined protocol (Table 3), we found no association between frequency of diarrhoea, vomiting, fever or cough in the past week and treatment protocol. This was true for both SAM and MAM admissions. There was also no association between the proportion of children admitted to hospital in the 4 months since discharge and treatment protocol.

**Table 3:** Morbidity at four months post-discharge.

	SAM cases				MAM cases			
	Standard protocol	Combined protocol	Adjusted difference	P value	Standard protocol	Combined protocol	Adjusted difference	P value
	%	%	(95% CI)		%	%	(95% CI)	
	n = 64	n = 73			n = 375	n = 268		
Diarrhoea in past week	10.9%	5.6%	-0.71 (-2.4, 1.0)	0.41	8.0%	9.2%	0.05 (-0.9, 1.0)	0.91
Vomiting in past week	7.8%	8.5%	0.09 (-1.2, 1.3)	0.89	4.8%	7.6%	0.21 (-1.4, 1.8)	0.80
Fever in past week	9.4%	9.9%	0.24 (-1.1, 1.5)	0.72	10.2%	12.6%	0.34 (-0.9, 1.6)	0.59
Cough in past week	23.4%	16.9%	-0.29 (-1.8, 1.3)	0.72	18.2%	14.9%	-0.41 (-1.7, 0.9)	0.53
Hospitalised in past 4 months	4.7%	5.6%	0.19 (-1.3, 1.7)	0.81	2.4%	3.8%	0.52 (-1.8, 2.9)	0.66

Logistic regression analysis, accounting for clustering. n = number of children.

As seen in Fig 1, 76% of children assessed at follow-up had repeatable BIA measurements that could be included in the analysis. There was no significant difference in body composition outcomes four months post-discharge between those treated with the combined and those treated with the standard protocol (Table 4 **& whole sample comparison in S2 Table in** S1 File). This includes FFM and fat mass levels (**S1 Fig** in S1 File), determined by BIA, as well as raw BIA outcomes (height-adjusted vectors, phase angle) (**S2 Fig in** S1 File) and skinfold thickness z-scores and ratio. Since this question was especially important for MAM cases, we present analysis for both groups as a whole and for MAM admissions only. Regarding adiposity levels at follow-up in general, 0.9% of the sample had triceps skinfold thickness above 2 z-scores, and 6.5% had a subscapular skinfold thickness above 2 z-score (see histograms in **S3 and S4 Figs in** S1 File). FFM comprised 73.5% of children’s body weight for SAM survivors and 72.7% for MAM survivors.

**Table 4:** Body composition at four-month follow-up.

	SAM cases				MAM cases			
	Standard protocol mean (SD)	Combined protocol mean (SD)	Adjusted difference	P value	Standard protocol mean (SD)	Combined protocol mean (SD)	Adjusted difference	P value
	n = 53	n = 56	(95% CI)*		n = 276	n = 198	(95% CI)*	
Fat free mass (kg) §	6.03 (0.68)	6.12 (0.62)	0.07 (-0.22, 0.35)	0.63	6.33 (0.78)	6.25 (0.89)	-0.10 (-0.31, 0.11)	0.37
Fat mass (kg) §	2.2 (0.68)	2.35 (0.94)	0.13 (-0.15, 0.42)	0.37	2.37 (0.86)	2.33 (0.97)	-0.07 (-0.33, 0.20)	0.63
R/h	1247.3 (182.9)	1223.4 (151.0)	-19.5 (-88.6, 49.5)	0.58	1195.3 (155.5)	1210.9 (162.6)	13.73 (-29.8, 57.2)	0.54
Xc/h	72.65 (16.1)	73.29 (16.1)	0.10 (-8.71, 8.92)	0.84	72.87 (15.8)	75.32 (21.2)	2.27 (-2.10, 6.65)	0.31
Phase angle °	3.46 (0.78)	3.55 (0.79)	0.09 (-0.20, 0.39)	0.98	3.48 (0.63)	3.55 (0.78)	0.05 (-0.14, 0.24)	0.58
Skinfold thickness ratio	1.14 (0.21)	1.07 (0.19)	-0.06 (-0.15, 0.02)	0.13	1.14 (0.21)	1.15 (0.22)	0.005 (-0.08, 0.09)	0.91
Tricep skinfold z score	-0.47 (1.43)	-0.34 (1.19)	0.04 (-0.96, 1.03)	0.94	-0.25 (1.25)	-0.25 (1.10)	-0.004 (-0.72, 0.72)	0.99
Subscap skinfold z-score	0.07 (1.46)	0.49 (1.17)	0.33 (-0.53, 1.20)	0.45	0.30 (1.20)	0.21 (1.19)	-0.06 (-0.80, 0.68)	0.88

n = number of children.

*adjusted for, age and sex; allowing for clustering. § fat-free and fat mass calculated using an equation from healthy children in The Gambia (17). Skinfold thickness ratio = tricep/subscapular. R/h = resistance index; Xc/h = reactance index.

### Relapse

We examined the proportion of children that relapsed following treatment (Table 5). For those successfully discharged as cured, 3% of SAM cases and 11% of MAM cases relapsed within four months. There was no significant difference in odds of relapse for those in the combined protocol and those in the standard protocol (Table 5).

**Table 5:** Relapse at four months post-discharge and comparison between treatment arms.

All follow-ups				Discharged as cured			
From SAM to SAM		From MAM to GAM		From SAM to SAM		From MAM to GAM	
Combined	Standard	Combined	Standard	Combined	Standard	Combined	Standard
4/71	6/64	33/261	50/373	1/34	1/34	21/218	39/318
(6%)	(9%)	(13%)	(13%)	(3%)	(3%)	(10%)	(12%)
OR = 0.52 p = 0.31		OR = 0.08 p = 0.78		OR = 1.0 p = 0.75		OR = 0.90 p = 0.34	

OR = odds ratio. SAM = severe acute malnutrition; MAM = moderate acute malnutrition; GAM = global acute malnutrition, which is a combination of moderate and severe.

We assessed whether food security score might be a useful predictor of relapse, however, we found no association (Table 6). Being female, having a lower MUAC at admission, having a lower MUAC at discharge, and having a lower weight gain per day were all significantly associated with odds of relapsing in the whole sample. For those who were discharged as cured, being female and having a lower MUAC at discharge remained positively associated with odds of relapsing (Table 6).

**Table 6:** Predictors of relapse at four months post-discharge.

	All follow-ups				Discharged as cured			
	Relapsed (GAM to GAM) N = 666	Didn’t Relapse N = 117	Odds ratio	P value	Relapsed (GAM to GAM) N = 534	Didn’t Relapse N = 73	Odds ratio	P value
	Mean (SD) or %	Mean (SD) or %	(95% CI)		Mean (SD) or %	Mean (SD) or %	(95% CI)	
**Food security score**	3.14 (3.1)	3.16 (3.1)	0.99 (0.9, 1.1)	0.97	2.94 (3.1)	3.04 (3.0)	0.99 (0.9, 1.1)	0.84
**Female Sex**	70.0%	58.0%	1.69 (1.0, 2.7)	0.03*	73.6%	57.3%	2.07 (1.1, 3.9)	0.03*
**MUAC at admission**	11.80 (0.5)	11.96 (0.4)	0.53 (0.4, 0.8)	0.001*	12.00 (0.3)	12.03 (0.4)	0.87 (0.5, 1.6)	0.66
**MUAC at discharge**	12.54 (0.6)	12.99 (0.5)	0.14 (0.1, 0.2)	<0.001*	12.81 (0.4)	13.05 (0.4)	0.11 (0.04, 0.3)	<0.001*
**Age at admission**	11.63 (9.0)	11.79 (6.4)	1.00 (0.8, 1.0)	0.81	12.37 (8.6)	12.21 (6.4)	1.00 (0.96, 1.0)	0.85
**Weight gain (g /kg/day)**	1.58 (1.2)	2.02 (1.3)	0.74 (0.6, 0.9)	0.001*	1.79 (1.3)	2.12 (1.3)	0.81 (0.7, 1.0)	0.05

MUAC = mid-upper arm circumference. Food security score was generated from the FAO Food Insecurity Experience Scale (FIES) questionnaire. GAM = global acute malnutrition, which includes both severe and moderate forms.

## Discussion

This follow-up study aimed to assess whether acutely malnourished children treated with a simplified, combined protocol have similar anthropometry, sustained recovery and morbidity risk as children treated with the standard protocol at four months post-discharge. We were particularly interested in outcomes of SAM children in the combined protocol who have, on average, a reduced dosage of RUTF compared to the standard protocol, and MAM children treated with RUTF in the combined protocol, rather than RUSF as per the standard protocol.

Despite the children in the combined protocol having more severe anthropometric deficits at admission, we found no significant difference in weight, height, MUAC, WHZ and WAZ at four months post-discharge between the two protocols. We did find that LAZ was significantly lower for MAM survivors in the combined protocol than the standard protocol, however this reflects significant differences that existed at admission as “change in LAZ” since admission was similar between the groups. Weight gain was also higher in the combined vs standard protocol, which is again likely due to greater severity of weight deficits at admission, and either faster catch-up or possibly some regression to the mean. Morbidity since discharge was also similar; importantly, this was likewise true for SAM children in the combined protocol who on average received a lower dosage of RUTF than those in the standard protocol. These outcomes are in line with the conclusions of the main trial which found non-inferior recovery between the combined and standard protocols (76.3% vs 73.5%, risk difference of .03 (95% CI -0.05 to 0.10, p = 0.52)) [8].

Body composition outcomes were also similar at four months post-discharge and MAM children treated with RUTF did not have significantly different amounts or ratios of adiposity than those treated with RUSF. The mean subcutaneous fat levels remained close to the WHO global norm (mean tricep = -0.28 z-score; mean subscapular = 0.25 z-scores) suggesting no evidence of excessive fat gain at this stage post-treatment. Catch-up in in fat mass after SAM recovery may be beneficial at mitigating high post-discharge mortality, and this may be at a cost of muscle mass gain, as seen in long-term SAM follow-up studies [21, 23, 24].

Relapse rates up until four months post-discharge were 3% for children admitted with SAM and 11% for children admitted with MAM, when restricting the analysis to those discharged as cured. If we include the whole sample (i.e. also those who defaulted treatment or who did not respond to treatment), relapse proportions were greater (8% for SAM and 13% for MAM), however it can be argued that this is not true relapse as children who defaulted or failed treatment may not have recovered to begin with. The reduced dosage in the combined protocol may have impacted relapse rates [25], however we found the odds of relapsing were similar between the two protocol arms. Another study with a reduced RUTF dosage regime also reported no impact on relapse rates compared to standard dosage in uncomplicated SAM cases in Burkina Faso (2.4% relapse vs 1.8%; *p* = 0.69) [26]. They did however find a small but significant negative effect on linear growth, which we did not find. Among those who recovered, being female and having a lower MUAC at discharge (mean MUAC 12.54 vs 12.99) were positively associated with odds of relapsing, suggesting that these children might benefit from more proactive follow-up at discharge. Comparing relapse rates with those in other studies is difficult due to varying follow-up periods and differing definitions for both the numerator and the denominator, as highlighted by a recent review [12]. Papers in that review with a follow-up period up to six months found SAM relapse rates between 1.9% and 17% [12, 27, 28]. Across the studies, the strongest, most consistent risk factor associated with relapse was having lower anthropometric measurements upon admission to and discharge from treatment, which is in line with one of our findings.

It is important to note that this was a semi-passive follow-up process, hence relapse rates may not be accurate. Participants were asked to attend their follow-up appointments at the clinic and were called or visited to encourage attendance if they missed their appointment. Loss to follow-up would likely have been lower if study measurements took place at participant’s homes. Budget constraints, which prevented follow-up of the final children enrolled between February and May 2018, as well as the two general elections that took place in 2017, also contributed to loss to follow-up. This limitation affects both the study arms equally and those lost to follow-up had similar baseline characteristics as the sample retained, although a small element of survivor bias may be present. Since children in this study were recruited based on MUAC definitions of wasting, these findings may not be generalisable to children diagnosed with SAM based on WHZ. Another limitation of this study is the short follow-up period. In trying to move towards a more standard definition of relapse, follow-up until 6 months post-discharge is recommended [12, 25]. Additionally, longer-term follow-up is needed to further explore effects of treatment on body composition and health.

## Conclusion

Acutely malnourished children treated with a simplified, combined protocol have similar sustained recovery and post-discharge morbidity risk as children treated with current standard care. SAM children treated with the combined protocol had similar anthropometry and relapse rates four months post-discharge to those treated with the standard protocol, despite reduced dosages of RUTF. MAM children treated with RUTF in the combined protocol had similar body composition to those treated with RUSF in the standard protocol and neither group exhibited excess adiposity at four months post-discharge. These results strengthen the conclusions of the main trial that a combined protocol is non-inferior in terms of recovery to standard care. Moving towards a simplified, combined approach could treat more children, stretch existing resources further, and contribute to achieving Sustainable Development Goal Two of ending all forms of malnutrition.

## Supporting information

S1 File (DOCX)
