# Supplementary material for: Relapse and post-discharge body composition of children treated for acute malnutrition using a simplified, combined protocol: A nested cohort from the ComPAS RCT
Source: PLoS One. 2021 Feb 3;16(2):e0245477. doi: 10.1371/journal.pone.0245477 (PMC7857614; doi:10.1371/journal.pone.0245477)
Supplement: S1 File — (DOCX) [file pone.0245477.s001.docx]

**Supplementary Material: Questionnaire**

|  | | Please **circle** your response, enter text, or enter integer |
| --- | --- | --- |
| **1.** | **Anthropometry** | **Weight in kg :**  (min 04.00, max 30.00)    **Height in cm:**  (min 025.0, max 135.0)    **MUAC in cm:**  (min 06.0, max 16.00) |
| **2.** | **Anthropometry**  Does the child have oedema? | (Circle one)  Yes  No |
| **3a** | **Skinfold thickness reading 1** | Tricep skinfold in mm  Subscapular skinfold in mm |
| **3b** | **Skinfold thickness reading 2** | Tricep skinfold in mm  Subscapular skinfold in mm |
| **4** | **Bioelectrical Impedance analysis (BIA)**  Reading 1 | Impedance at 50 khz  Resistance at 50 khz  Reactance at 50 khz  Phase angle at 50 khz |

| **5** | **Bioelectrical Impedance analysis (BIA)**  Reading 2 | | Impedance at 50 khz  Resistance at 50 khz  Reactance at 50 khz  Phase angle at 50 khz |
| --- | --- | --- | --- |
| **6a.** | **Medical History - careseeking**  In the past week, has the child visited a medical clinic or hospital? | | (Circle one)  Yes  No  Don’t know |
|  | **6b.** | **Medical History-Diarrhea**  In the past week, did the child suffer from diarrhea (watery stool 3 or more times)? | (Circle one)  Yes  No🡪 go to 5c  Don’t know🡪 go to 5c |
|  | **6c.** | If yes, how many days has the child suffered from diarrhea? | (Enter days 1-7, 9 if don’t know) |
|  | **6d.** | **Medical History-Vomiting**  In the past week, did the child suffer from vomiting? | (Circle one)  Yes  No🡪 go to 3e  Don’t know🡪 go to 3e |
|  | **6e.** | If yes, how many days has the child suffered from vomiting? | (Enter days 1-7, 9 if don’t know) |
|  | **6f.** | **Medical History-Fever**  In the past week, did the child suffer from fever? | (Circle one)  Yes  No🡪 go to 3g  Don’t know🡪 go to 3g |

|  | **6g.** | If yes, how many days has the child suffered from fever? | (Enter days 1-7, 9 if don’t know) |
| --- | --- | --- | --- |
|  | **6h.** | **Medical History-Cough**  In the past week, did the child suffer from cough? | (Circle one)  Yes  No🡪 go to 4  Don’t know🡪 go to 4 |
|  | **6i.** | If yes, how many days has the child suffered from cough? | (Enter days 1-7, 9 if don’t know) |
|  | **6j.** | **Medical History - other**  In the past week, has the child had any other illness? | (Circle one)  Yes (please specify) ____________________  No  Don’t know |
| **7.** | **Medical History**  Has the child been in a nutrition treatment program in the last 4 months? | | (Circle one)  Yes  No  Don’t know |
|  | **7a.** | **Medical History**  Has the child been hospitalized in the last 4 months? | (Circle one)  Yes  No  Don’t know |
|  | **7b.** | **Medical History**  Is the child currently taking any medication? | (Circle one)  Yes  No  Don’t know |
|  | **7c.** | **Medical History**  Was this child breastfed in the last 24 hours? | (Circle one)  Yes  No  Don’t know |
| **8.** | **Household Food Security**  In the last month, has the household received food distribution from the government NGOs? | | (Choose one)  Yes  No  Don’t know |
| **8a** | During the past 12 months, were you worried about not having enough food to eat because of lack of money? | | (Choose one)  Yes  No  Don’t know |
| **8b** | During the past 12 months, was there a time when you were unable to eat healthy and nutritious food because of lack of money? | | (Choose one)  Yes  No  Don’t know |
| **8c** | During the past 12 months, was there a time when you ate only a few kinds of foods because of lack of money? | | (Choose one)  Yes  No  Don’t know |
| **8d** | During the past 12 months, was there a time that you had to skip a meal because of lack of money? | | (Choose one)  Yes  No  Don’t know |
| **8e** | During the past 12 months, was there a time you ate less than you thought you should? | | (Choose one)  Yes  No  Don’t know |
| **8f** | During the past 12 months, was there a time that your household ran out of food, because of lack of money? | | (Choose one)  Yes  No  Don’t know |
| **8g** | During the past 12 months, was there a time when you were hungry but did not eat, because of lack of money? | | (Choose one)  Yes  No  Don’t know |
| **8h** | During the past 12 months, was there a time that you went without food for a whole day because of lack or money? | | (Choose one)  Yes  No  Don’t know |

**S1 Table: Differences at baseline between those lost to follow-up and those retained**

|  | **Retain in the sample**  **N= 780** | | | **Lost to follow-up**  **N=1123** | | |
| --- | --- | --- | --- | --- | --- | --- |
|  | Total | Combined  Protocol | Standard  Protocol | Total | Combined  protocol | Standard  protocol |
| Median age at admission  (months) | 10  (IQR 8-14) | 9  (IQR 7-14) | 10  (IQR 8-14) | 10  (IQR 8-14) | 10  (IQR 8-14) | 9  (IQR 8-14) |
| Males | 39% | 38% | 42% | 36% | 37% | 36% |
| Weight at admission (kg) | 6.9  (1.2) | 6.9  (1.2) | 7.1  (1.2) | 6.8  (1.2) | 6.7  (1.3) | 6.9  (1.2) |
| Height at admission  (cm) | 70.6  (6.3) | 70.4  (7.6) | 70.7  (6.7) | 69.3  (6.4) | 69.2 (6.4) | 69.4 (6.3) |
| MUAC at admission  (cm) | 12.0  (0.5) | 12.0 (0.6) | 12.0 (0.5) | 11.8  (0.5) | 11.7 (0.6) | 11.8 (0.5) |
| Oedema at admission | 0.5% | 0.8% | 0.2% | 1.2% | 1.7% | 0.5% |
| MUAC at last measurement | 13.0 (0.5) | 13.0 (0.5) | 13.0 (0.5) | 12.4 (0.7) | 12.4 (0.7) | 12.5 (0.7) |
| Died | 0 | 0 | 0 | 2.3% | 2.1% | 2.5% |
| Discharged as recovered | 78.8% | 76.1% | 80.7% | 36.7% | 37.4% | 35.9% |

**S2 Table: Body composition at 4 month follow-up for whole sample**

|  | Whole sample (both SAM and MAM admissions) | | | |
| --- | --- | --- | --- | --- |
|  | Standard protocol  mean (SD)  n=353 | Combined protocol  mean (SD)  n=288 | Adjusted  difference  (95% CI)* | P value |
| Fat free mass (kg) § | 6.32 (0.81) | 6.30 (0.91) | -0.04 (-0.27, 0.18) | 0.70 |
| Fat mass (kg) § | 2.40 (0.87) | 2.41 (0.97) | -0.04 (-0.25, 0.17) | 0.71 |
| R/h | 1201.2 (160.7) | 1205.7 (161.8) | 5.42 (-38.2, 49.1) | 0.81 |
| Xc/h | 72.83 (15.5) | 75.12 (20.9) | 1.86 (-2.65, 6.36) | 0.42 |
| Phase angle ° | 3.49 (0.65) | 3.57 (0.77) | 0.05 (-0.16, 0.26) | 0.62 |
| Skinfold thickness ratio | 1.15 (0.21) | 1.14 (0.21) | -0.007 (-0.08, 0.07) | 0.85 |
| Tricep skinfold z score | -0.29 (1.26) | -0.26 (1.09) | 0.02 (-0.71, 0.74) | 0.97 |
| Subscap skinfold z-score | 0.24 (1.24) | 0.27 (1.16) | 0.03 (-0.69, 0.74) | 0.94 |

**S1 Figure: Fat mass vs fat-free mass index for intervention and control groups**

**
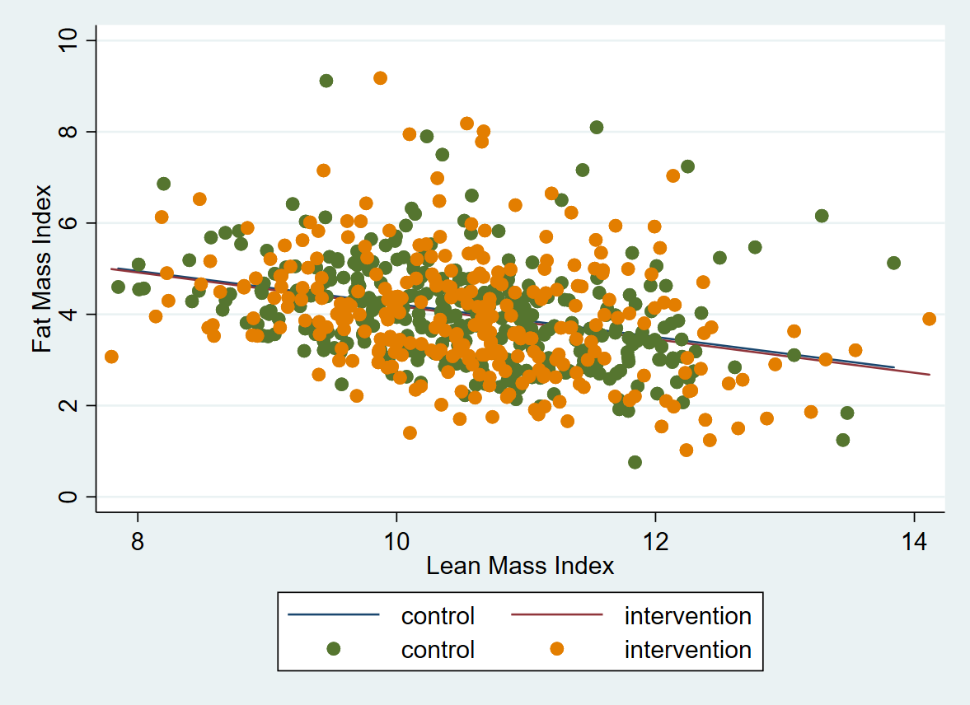
**

**S2 Figure: Graphs showing over-lapping vector ellipses for BIA outcomes for intervention and control groups**
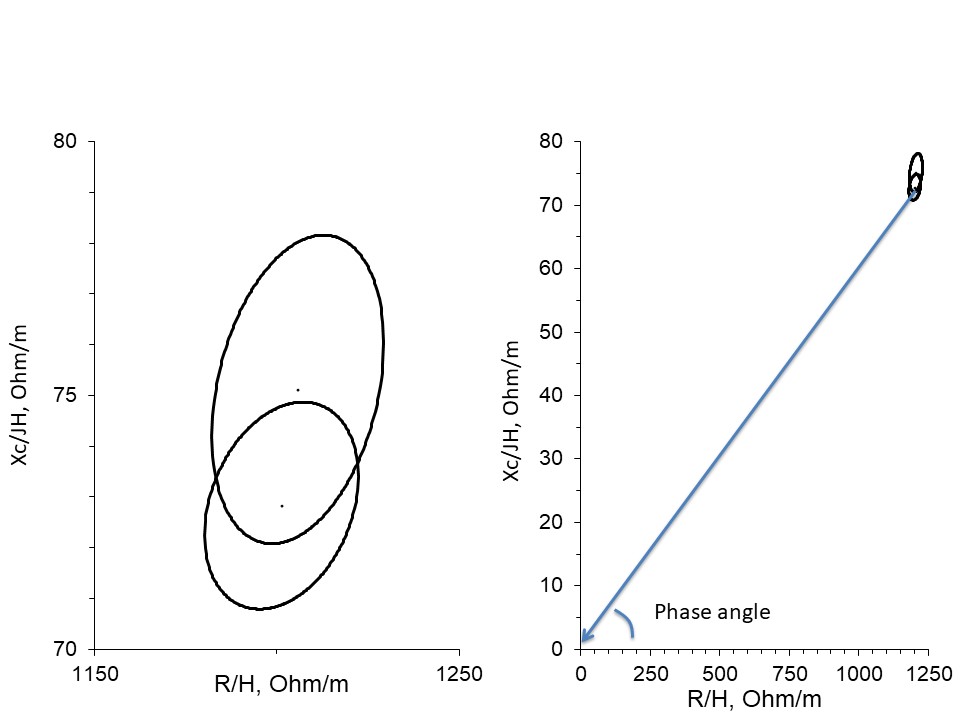


*Xc= reactance; R=resistance

**S3 Figure: Histograms of tricep skinfold thickness for intervention and control groups**

**
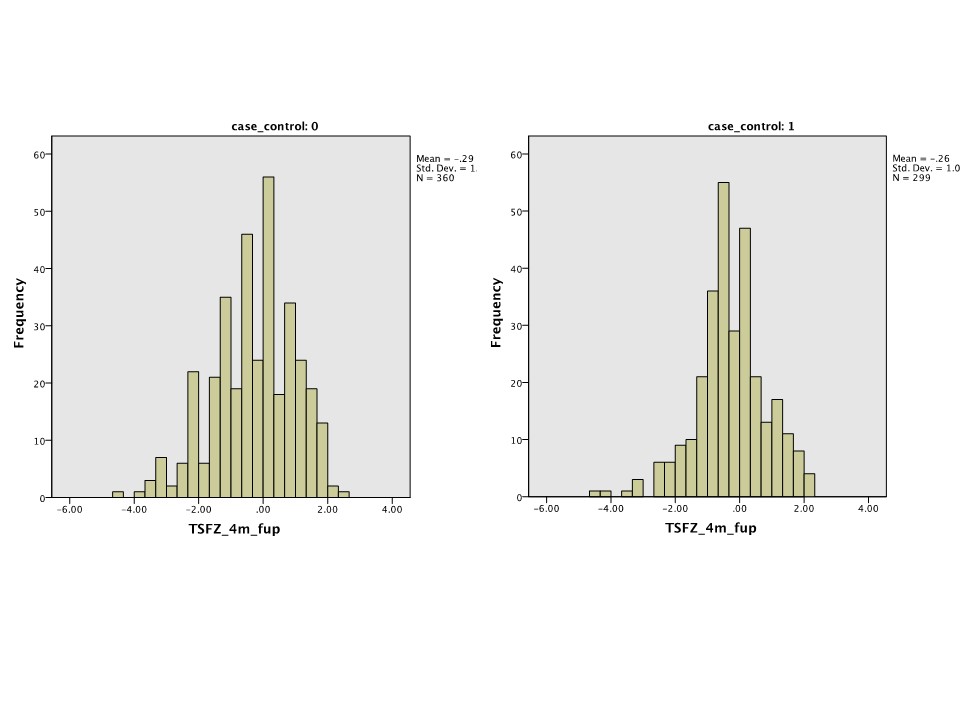
**

*0 (left graph)= control group; 1 (right graph)= intervention group

**S4 Figure: Histograms of subscapular skinfold thickness for intervention and control groups**

**
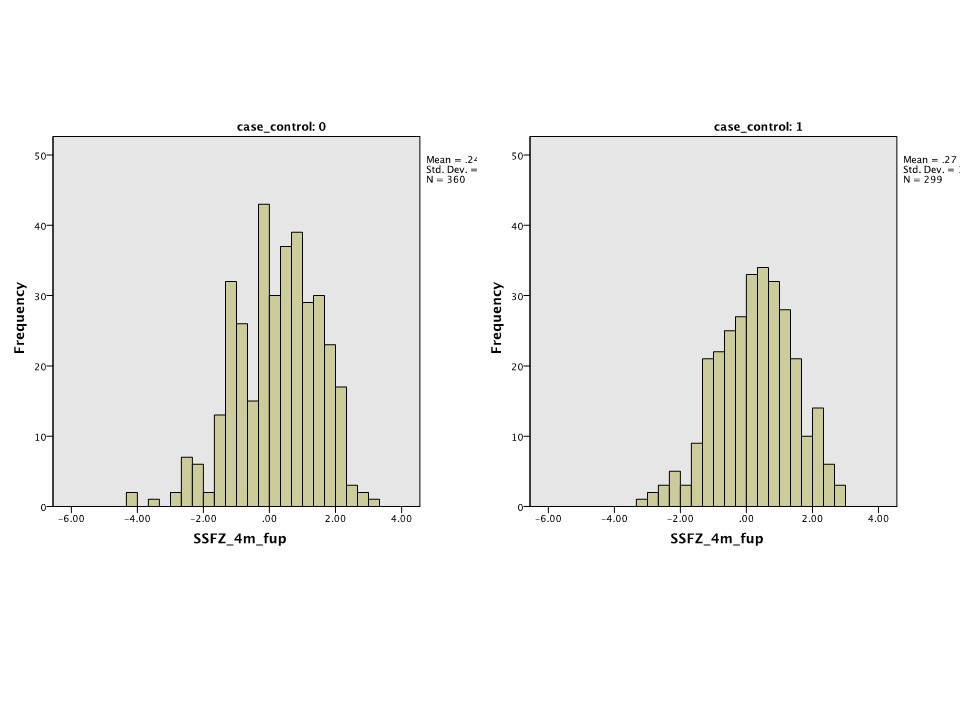
**

*0 (left graph)= control group; 1 (right graph)= intervention group
